# Effect of Shear and pH on Heat-Induced Changes in Faba Bean Proteins

**DOI:** 10.3390/foods14162906

**Published:** 2025-08-21

**Authors:** Rui Yu, Thom Huppertz, Todor Vasiljevic

**Affiliations:** 1 Advanced Food Systems Research Unit, Institute for Sustainable Industries and Liveable Cities, College of Sports, Health and Engineering, Victoria University, Melbourne, VIC 8001, Australia; rui.yu3@live.vu.edu.au (R.Y.);; 2 University College Cork, T12 K8AF Cork, Ireland

**Keywords:** faba bean protein isolate, globulin, thermal processing, protein concentration, pH, shear, solubility, secondary structure, protein aggregation

## Abstract

Commercially relevant processing conditions, including protein concentration, pH and shearing and their impact on the solubility, heat stability, and secondary structure of faba bean proteins (FBPIs), were studied. Most of the examined properties, including protein solubility and heat stability, were due to the simultaneous effects of pH and concentration. The shearing rate played a crucial role in determining the heat stability of FBPI during thermal processing through protein molecular activities, such as inter- and/or intramolecular force interactions. Under the heat treatment conditions (temperature of 95 °C and time of 30 min), the shearing rate of 1000 s^−1^ enhanced the heat stability, compared to 100 s^−1^. Meanwhile, concentration and pH shift contributed to the conformation of various protein structures of faba bean protein isolates. This study revealed that these structural changes involve the unfolding of the protein’s native tertiary structure, which likely exposes hydrophobic and sulfhydryl (–SH) groups, ultimately leading to protein aggregation. It also provided a comprehensive understanding of faba bean protein functionality by studying various interactions of FBPI proteins under thermal processing systems.

## 1. Introduction

Grain legumes, such as beans and lentils, offer a highly beneficial nutritional profile for human consumption. They are low in fat and rich in protein, dietary fibre, iron, zinc, and essential vitamins, including folate, riboflavin, and thiamine. Additionally, grain legumes contain antioxidants and various bioactive compounds that provide substantial health benefits [1,2]. The consumption of grain legumes has been linked to numerous health advantages, such as a reduced risk of colorectal cancer [3], improved gut health, lowered blood cholesterol levels [4], and a decreased risk of cardiovascular disease [5].

Faba bean protein isolate (FBPI) is particularly rich in essential amino acids, including branched-chain amino acids (BCAAs) and lysine, which is considered the limiting amino acid in cereal grains [6]. However, the use of legume protein as a food ingredient has been limited mainly due to challenges related to poor protein solubility [7]. Legume proteins primarily consist of two types: globulins, which make up about 70–80%, and albumins, which account for approximately 10–20%. The solubility of these proteins is affected by the solution composition, including factors like temperature, ionic strength, pH, and protein concentration [8] and by environmental stresses, such as thermal treatment.

Thermal processing, such as pasteurisation and sterilisation, is commonly used to treat liquid food products to improve food safety and extend product shelf life. Heat application can affect the properties of legume proteins by inducing aggregation, precipitation, or gelling, a phenomenon that is influenced by the intensity of the heating process and the heat stability of the proteins involved [9]. Previous studies have shown that concentration, pH, ionic strength, shearing rate, and the presence of certain additives are major environmental factors affecting protein structure [7,10,11,12,13]. Proteins may unfold and obtain various conformational structures during the heating process, including flexible strands, fibrils, and nano- and microparticles [14,15]. Notably, varying concentrations of protein can significantly impact protein–protein interactions, leading to variations in solubility and aggregation characteristics [16]. Recent studies have demonstrated that a higher concentration of pea proteins during heat treatment can result in larger aggregate formation and enhanced hardness of gels [17,18]. pH not only affects the ability of globulins to preferentially complex with ions but also induces the formation of soluble ternary complexes [19]. In addition, liquid foods are subjected to various mechanical forces, such as hydrodynamic shear, during commercial processing operations [20]. These mechanical forces occur at multiple stages of thermal processing, including pumping, stirring, spraying, homogenisation, and passage through heat exchangers. Such mechanical forces may lead to reversible changes in food products’ structural and functional properties [21]. As Wang et al. [13] demonstrated, an increased shearing rate can contribute to an increase in surface hydrophobicity and conformational flexibility, which enhances electrostatic repulsion, decreases particle size, and leads to the formation of a loose and porous microstructure [13]. Hence, the shearing rate is expected to cause a change in the size of the soluble fraction and the rate of destabilisation.

Previous studies involving faba bean proteins have applied heat treatment at temperatures ranging from 20 to 80 °C to pulse proteins, simulating certain industry processes [22,23,24]. This temperature range has a marginal impact on most of these proteins. Also, denaturation of the protein is necessary for the subsequent aggregation of denatured molecules and the creation of the faba bean denaturation kinetics model [25,26]. Hall and Moraru [27] illustrated that at a low protein concentration of 1.5% (*w*/*w*), the denaturation temperature of faba bean protein concentrate was between 89 and 94 °C. Generally, previous studies have investigated faba bean protein molecular activities at shear rates between 1 and 1000 s^−1^ and heat treatments ranging from 0.5 min to 30 min [19,25,27,28], or examined heated protein dispersions with no or low shearing [29,30]. However, there is limited understanding of the denaturation behaviour of FBPI under prolonged thermal processing with various shear rates at different protein concentrations and pH. To gain more insight into the properties of faba bean proteins during thermal aggregation, this study aimed to adopt a more comprehensive approach. It is based on our previous research that established the optimum conditions for hydration of faba bean protein isolate [8]. The pH levels of 6.8 and 7.5 were optimal for achieving high solubility of faba bean protein isolate in the past [8]. Besides the optimal pH for proper hydration, shear rates of 100 s^−1^ and 1000 s^−1^ were selected to examine their effect on protein behaviour during denaturation, especially since high shear rates are experienced during pumping and homogenisation [17]. The concentrations chosen, 4% and 8% (*w*/*w*), are commonly used in the preparation of protein-based beverages, making it essential to understand how concentration affects protein behaviour, solubility, and functionality. Different concentrations can significantly influence protein–protein interactions, leading to variations in solubility and aggregation characteristics, which are crucial for determining optimal processing conditions [31]. In practical applications, protein concentrations typically stay within certain ranges, making it useful to analyse these values to inform product formulations and guide commercial use. Examining different interactions and properties of proteins helps determine solubility thresholds that produce desirable functions like emulsification, foaming, and gelling. This ensures resources are used efficiently and production remains cost-effective.

## 2. Materials and Methods

The FBPI powder with a protein content of 88% (*w*/*w*, dry basis) was kindly provided by Australian Plant Proteins (Horsham, Victoria, Australia). According to the product specification, the FBPI powder also contained around 2–3% ash, 5–6% fat, 0.5% carbohydrates, and 3–4% moisture. All chemicals used in this study were of analytical grade and were obtained from Sigma-Aldrich Pty Ltd. (Castle Hill, NSW, Australia) unless stated otherwise. All experiments were conducted using Milli-Q water (Merck Millipore, Bayswater, VIC, Australia).

### 2.1. Sample Preparation

The 4% or 8% *w*/*w* faba bean protein dispersions were prepared by dispersing an appropriate amount of the FBPI powder in Milli Q water prewarmed to 65 °C and pH adjusted to a required level. Upon addition, the dispersion was stirred at 1000 rpm using an overhead stirrer (ISG, Westlab PTY Ltd., Ballarat, Australia) in a water bath at 65 °C for 30 min. The pH was subsequently adjusted to 6.8 or 7.5 using 1 M NaOH or HCl. The resultant four different dispersions were heated at 95°C for 30 min under constant shear rates of 100 or 1000 s^−1^, without altering the protein concentration or pH.

### 2.2. Determination of Protein Solubility

Upon the completion of the sample preparation procedure, the solubility of the protein in all samples was measured after centrifuging (Model J2HS; Beckman, Fullerton, CA, USA) at 700 g and 20 °C for 10 min. The solubility was quantitatively expressed as prescribed by Dissanayake et al. (2012) as an amount of protein content remaining in the supernatant relative to that of the original dispersion [32]:

$$Solubility\;(\%)=\frac{Protein\;content\;of\;supernatant}{Protein\;content\;of\;the\;original\;dispersion}\cdot 100$$

The protein content in the bulk and the supernatant was determined following the Kjeldahl method.

### 2.3. Heat Treatment

After preparation, the samples were split into two parts and subjected to a heat treatment at 95 °C for 30 min under a constant shear rate (either 100 or 1000 s^−1^). This was performed using a pressure cell (CC25/PR-150) on a rheometer (MCR 302e, Anton Paar GmbH, Graz, Austria) with a steady pressure of 0.556 kPa, following the method described in Mediwaththe [33].

### 2.4. Determination of Heat Stability

The heat stability of proteins in the supernatant of FBPI dispersions was evaluated in duplicate based on their protein solubility following each heat treatment, using the procedure outlined in Section 2.3. Heat stability, or more precisely, the solubility after heat treatment, was expressed with the equation provided below.

$$Heat\;stability\;(\%)\;=\frac{Protein\;content\;of\;supernatant}{Protein\;content\;of\;the\;untreated\;supernatant}\cdot 10$$

### 2.5. Zeta Potential and Particle Size

Zeta potential and particle size measurements were carried out at least in triplicate readings using a Zetasizer Nano ZS from Malvern Instruments (Worcestershire, UK), following the protocol outlined by Yu et al. [8]. Before and after treatment, all samples were diluted 1:200 times using Milli Q water at 20 °C.

### 2.6. Attenuated Total Reflectance Fourier Transform Infrared Spectroscopic (ATR-FTIR) Analysis of FBPI Dispersions

The alterations in protein secondary structure were analysed utilising a PerkinElmer Frontier FTIR Spectrometer (Frontier 1, PerkinElmer, Waltham, MA, USA), equipped with a built-in attenuated total reflection (ATR) accessory. The protein dispersion samples were loaded by same volume on the top-plate. Spectra were recorded at approximately 20 °C within three hours after treatment, averaging 16 scans with a resolution of 4 cm^−1^, following background subtraction [34]. The second derivative of all FTIR spectra within the Amide I region (1700–1600 cm^−1^) was computed using Spekwin32-Spectragryph software (version 1.2.16) to improve resolution for qualitative assessment. Fourier self-deconvolution (FSD) and baseline correction were performed using Origin Pro 2024 software to pinpoint the significant peaks indicative of protein secondary structures in the Amide I region. Subsequently, peak fitting was executed using the Gaussian function and peak fitting methodology. This iterative approach refined the fitting process, allowing for the calculation of peak areas associated with α-helices, β-sheets, β-turns, β-sheets, and random Coil [35,36]. Statistical analysis was subsequently conducted in accordance with the specified guidelines.

### 2.7. Sodium Dodecyl Sulphide Polyacrylamide Gel Electrophoresis (SDS PAGE)

The SDS-PAGE was performed after treatment by combining heated samples, control samples, and centrifuged supernatants with SDS sample buffer [17]. They were prepared by diluting a 4 or 8% (*w*/*w*) protein dispersion to a final concentration of 1.0 mg/mL. Reducing samples were reduced by β-Mercaptoethanol. SeeBlue Plus2 Pre-stained Protein Standard (Thermo Fisher Scientific, Scorescby, VIC, Australia) was used as the molecular weight markers. Both non-reducing and reducing SDS-PAGE analyses were performed using standardised protocols, and gel images were obtained with Image Lab 5.1 software. The protein intensity in the treated samples, as visualised through reducing gel electrophoresis, was quantified as a percentage in relation to the control bulk dispersions.

### 2.8. Statistics

This study was carried out using a randomised block design (split-plot) and the General Linear Model Univariate function of SAS v.9.1. The concentration served as the main plot, while pH, heat treatment, and shearing rate were included as subplots. The replications served as a block. The entire experiment was replicated on multiple occasions (at least 3 independent times) to ensure the reliability and reproducibility of the data. The sample analyses were performed in triplicate. The level of significance was preset at *p* < 0.05.

## 3. Results

### 3.1. Solubility and Heat Stability

The solubility and heat stability of FBPI were influenced by pH, concentration, and shear rate. The experiments were conducted under optimal rehydration conditions established previously, resulting in all samples exhibiting a solubility exceeding 97%. This finding aligns with the results obtained in our previous study [8] (Table 1). While differences in solubility were observed as a function of protein concentration and pH, they were not statistically significant (*p* > 0.05).

On the other hand, shearing rate had a significant effect as the highest solubility and heat stability were observed at 1000 s^−1^ regardless of the concentration and pH level (Table 1). Faba bean protein isolate’s heat stability exceeded 95% across reconstitution levels, especially at 1000 s^−1^ shear rate, where it neared 100%. The pH of 6.8 yielded higher stability than a pH of 7.5 at this shear. At 100 s^−1^, the increasing concentration from 4% to 8% enhanced the heat stability at each pH level.

Furthermore, all other heated FBPI dispersions tested at 100 s^−1^ exhibited a significant decrease (*p* < 0.05) of approximately 3–4% in the amount of the total soluble protein following the same treatment. Consequently, when the shearing rate increased from 100 to 1000 s^−1^, heat stability was improved. Also, concentration appears to play a crucial role in the heat stability of FBPI. Specifically, the heat stability of protein at the 8% concentration was greater across all pH levels. Additionally, the variation in heat stability at the same concentration level was less pronounced among different pH values.

### 3.2. Zeta Particle and Particle Size

Generally, the average particle size of the bulk and serum at a pH of 6.8 was larger than that at a pH of 7.5, reflecting the reconstitution conditions. The sample with or without heat treatment was primarily influenced by pH (Table 2). For the control sample, the particle size of the bulk protein dispersion at a pH of 6.8 increased from 491 nm to 754 nm as the protein concentration rose from 4% to 8%. Meanwhile, the particle size of the supernatant at a pH of 6.8 was approximately 438 nm at 4%, which was similar to that of the bulk sample. In contrast, the supernatant at a pH of 6.8 at 8% was about 575 nm, which was smaller than that in the bulk dispersion. As Table 2 shows, the particle size of the bulk and supernatant control samples at a pH of 7.5 was consistent at approximately 195 and 292 nm, respectively, from 4% to 8%.

After the heat treatment, the average particle size of the bulk decreased at a pH of 6.8, depending on the applied shear. At 4% concentration, the average particle size declined significantly (*p* < 0.05) from 491 to 262 and 273 nm with shearing rates of 100 and 1000 s^−1^ (Table 2). At 8%, the particle size of bulk dispersions slightly (*p* > 0.05) decreased under a shearing rate of 100 s^−1^, from 754 to 672 nm, and significantly (*p* < 0.05) decreased to 457 nm at 1000 s^−1^ (Table 2). In contrast, the particle size of the bulk samples at a pH of 7.5 remained fairly stable and was only slightly affected (Table 2). Therefore, thermal processing had less impact on the particle size at higher protein concentrations.

Furthermore, the zeta potential of faba bean proteins (both bulk and supernatant) also changed depending on the processing conditions (Table 2). In alignment with our previous study, the negative charge at a pH of 7.5 was greater than that at a pH of 6.8 [8]. At 4% concentration, the zeta potential of the control bulk sample at a pH of 6.8 and a pH of 7.5 was −21.4 and −23.7 mV, respectively. When the concentration increased to 8%, the difference in zeta potential of the control bulk sample between a pH of 6.8 and pH of 7.5 was broadened, with values of approximately −21.8 mV and −32.5 mV, respectively (Table 2). Hence, the change in zeta potential of the dispersion at both a pH of 6.8 and 7.5 significantly decreased (*p* < 0.05) with increasing concentration. Specifically, the zeta potential at a pH of 7.5 became more negatively charged compared to that at a pH of 6.8. Additionally, the surface charge of the bulk sample at shearing rates of 100 s^−1^ and 1000 s^−1^ was influenced by both pH and concentration. Similar to the control sample, the zeta potential of the heated sample at a pH of 6.8 was higher than that at a pH of 7.5. Meanwhile, the zeta potential decreased with the rising concentration. In particular, the charge of bulk sample at a pH of 6.8, shearing rate 100 s^−1^, increased from −21.6 to −23.2 mV (Table 2) with concentration increasing from 4% to 8%. It was similar to the bulk sample at 1000 s^−1^, which elevated from −22.0 to −23.4 mV. At a pH of 7.5, the charge of the bulk sample at shearing rates of 100 and 1000 s^−1^ increased from −26.7 to −36.7 mV and −26.0 to −36.6 mV, respectively, with a rising concentration from 4% to 8%. Thus, the pH and concentration affect the zeta potential of FBPI dispersion. The impact of the shearing rate was dependent on the pH and concentration. When the processing environment was alkaline, the protein charge increased more with rising shearing rate.

### 3.3. Changes in the Secondary Structure of Proteins as Impacted by Processing Conditions

The changes in the secondary structure of proteins were studied by analysing FTIR spectra of all FBPI dispersions in the Amide I region. Peaks observed at different wavenumbers were assigned to specific secondary structures: intermolecular β-sheets (IEM β-S; 1612–1630 cm^−1^), β-sheet elements (BH; 1630–1644 cm^−1^), random coil (RC; 1645–1650 cm^−1^), α-helix (α-H; 1651–1664 cm^−1^), β turns (βT; 1665–1684 cm^−1^), intramolecular β-sheets (IAM β-S; 1685–1690 cm^−1^), and aggregated β-sheets (A β-S; 1691–1700 cm^−1^) [37,38,39].

**Figure 1:**
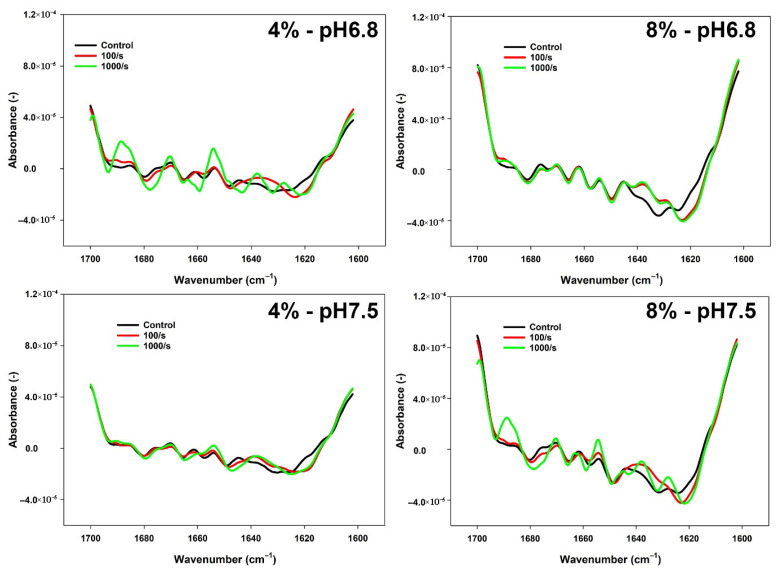
FTIR spectra of 4 or 8% FBPI dispersions held under different pH (6.8 or 7.5) subjected to a heat treatment (95 °C 30 min) and shearing (100 or 1000 s^−1^). The controls were not heated or sheared.

As Figure 1 illustrates, the secondary structure of the protein changes in various ways under different reconstitution conditions. To examine these conformational fluctuations, changes in the proportion of relevant structural elements in samples were quantified. According to Table 3, the secondary structure of the control FBPI dispersion has a significant difference under various reconstitution conditions. Specifically, the percentage of intermolecular β-sheets and β-sheets elements was higher at a pH of 6.8 than at a pH of 7.5 under the same concentration. At a pH of 6.8, both intermolecular β-sheet and β-sheet elements at 4% concentration showed the highest percentage, which was around 20.4% and 25%, respectively. As the concentration increased to 8%, the percentage of intermolecular β-sheets and β-sheets elements decreased to 13.5% and 12.4%, respectively (*p* < 0.05) (Table 3). In contrast, the intramolecular β-sheets were at the lowest value at 4% concentration, around 6.9% (Table 3). It increased dramatically to 16.7% (*p* < 0.05) when the concentration was 8%. At a pH of 7.5, the percentage of β-sheet elements decreased from 15.9% to 10.7% (*p* < 0.05) (Table 3). Notably, the percentage of the other secondary structures was fairly consistent.

Additionally, the proportion of random coil and α-helix content remained relatively stable under various reconstitution conditions (Table 3). In this regard, the intermolecular β-sheet and β-sheet elements were enhanced at 4% concentration or/and a pH of 6.8, while the intramolecular β-sheets were reduced simultaneously. Therefore, both pH and concentration influence the structural changes in faba bean protein isolate before heat treatment.

Compared to a shearing rate of 100 s^−1^, the shearing rate of 1000 s^−1^ has a greater impact on the change in protein secondary structure among heated samples. At a pH of 6.8, the proportion of β-sheet elements at 4% concentration significantly declined from 24.6% to 8.1% (Table 3). In contrast, the percentage of β-turns increased from 12.7% to 21.5% (*p* < 0.05) (Table 3). When the concentration was increased to 8%, the percentage of secondary structure elements remained steady across the varying shearing rates.

At a pH of 7.5, most secondary structures at 4% concentration also showed less change with the increasing shearing rate. However, the intramolecular and aggregated β-sheets significantly decreased from 18.1% to 14.1% and from 11.3% to 8%, respectively (*p* < 0.05), with the rising shearing rate (Table 3). As the concentration elevated to 8%, both the inter- and intramolecular β-sheet contents decreased from 17.1 to 12.6% and from 14.1% to 11.7%, respectively (*p* < 0.05). On the contrary, the percentage of α-helix, β turn, and aggregated β-sheets increased from 10.3 to 12.1%, 11.1 to 14.3%, and 10.1 to 13.8%, respectively (*p* < 0.05). Interestingly, the percentage of β-sheet elements remained stable across the varying shearing rates. Hence, at a concentration of 4%, the shearing rate induced a greater conformational change at a pH of 6.8. When the concentration was elevated to 8%, more change can be observed at a pH of 7.5.

### 3.4. Protein Partitioning

**Figure 2:**
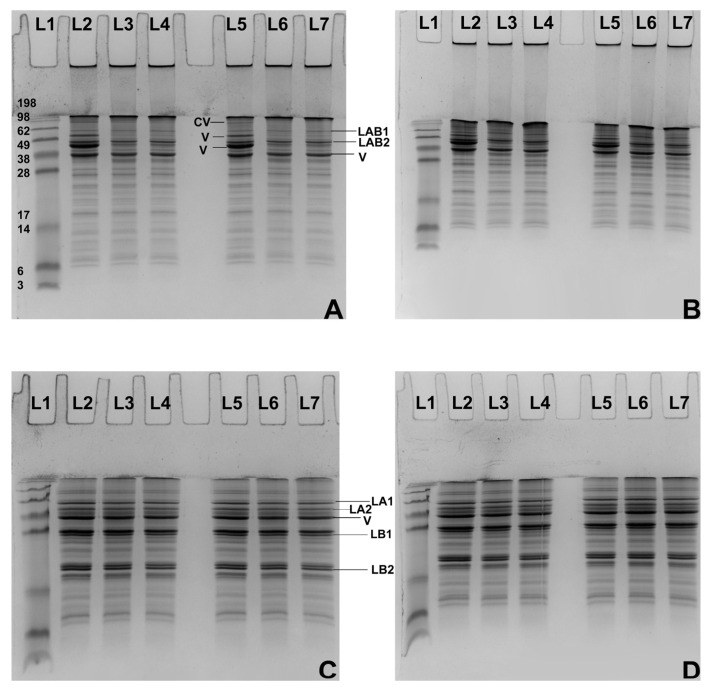
Non-reducing (NR) SDS-PAGE analysis of 4% (*w*/*w*) FBPI dispersions (bulk) and supernatant at a pH of 6.8 (**A**) or 7.5 (**B**) and reducing those of samples at a pH of 6.8 (**C**) or 7.5 (**D**). Lanes are designated as L1 molecular weight markers, L2 control bulk sample, L3 bulk sample heated 95 °C 30 min 100 s^−1^, L4 bulk sample heated 95 °C 30 min 1000 s^−1^, L5 control supernatant sample, L6 supernatant sample heated 95 °C 30 min 100 s^−1^, L7 supernatant sample heated 95 °C 30 min 1000 s^−1^. CV (convicilin), LAB1 (legumin A-B1), LAB2 (legumin A-B2), V (vicilin), LA1 (legumin acidic subunit 1), LA2 (legumin acidic subunit 2), LB1 (legumin basic subunit 1), and LB2 (legumin basic subunit 2).

**Figure 3:**
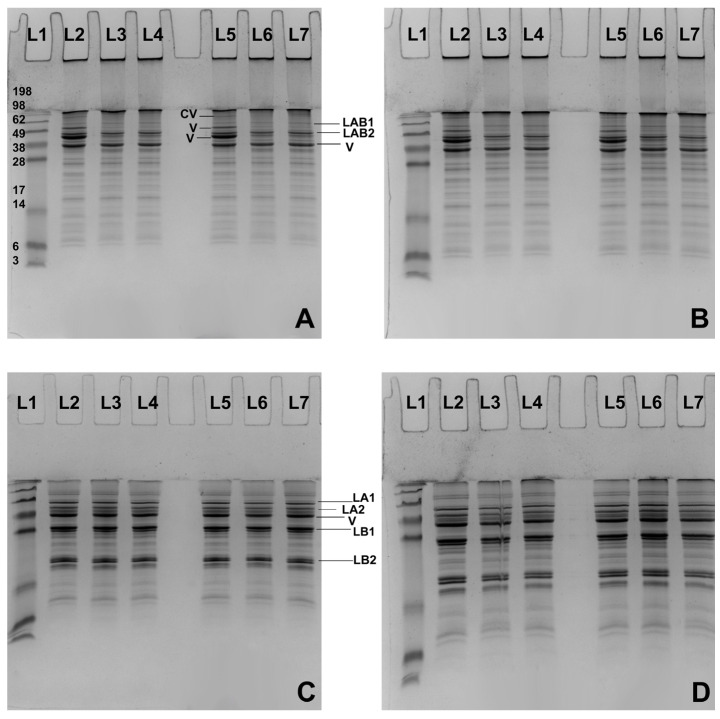
Non-reducing (NR) SDS-PAGE analysis of 8% (*w*/*w*) FBPI dispersions (bulk) and supernatant at a pH of 6.8 (**A**) or 7.5 (**B**) and reducing those of samples at a pH of 6.8 (**C**) or 7.5 (**D**). Lanes are designated as L1 molecular weight markers, L2 control bulk sample, L3 bulk sample heated 95 °C 30 min 100 s^−1^, L4 bulk sample heated 95 °C 30 min 1000 s^−1^, L5 control supernatant sample, L6 supernatant sample heated 95 °C 30 min 100 s^−1^, L7 supernatant sample heated 95 °C 30 min 1000 s^−1^. CV (convicilin), LAB1 (legumin A-B1), LAB2 (legumin A-B2), V (vicilin), LA1 (legumin acidic subunit 1), LA2 (legumin acidic subunit 2), LB1 (legumin basic subunit 1), and LB2 (legumin basic subunit 2).

The SDS-PAGE analysis revealed changes in the types and molecular weight distributions of soluble proteins solubilised in the different suspensions. Figure 2 and Figure 3 illustrate that faba bean proteins consist of several components, primarily identified as legumin and vicilin at 4% and 8% concentrations, respectively. Generally, the distribution of protein bands from the faba bean protein isolate changed consistently across different concentrations.

Additionally, two types of legumins can be recognised: legumin A-B1 and A-B2, which appear at approximately 56 kDa and 46 kDa, respectively, when comparing reducing and non-reducing PAGE (Figure 2 and Figure 3). Under reducing conditions, three prominent protein bands at the top of the well disappeared, resulting in the emergence of four new distinct bands at 44, 34, 26, and 20 kDa, identified as α-legumin 1, α-legumin 2, β-legumin 1, and β-legumin 2, respectively [8,40] Moreover, the bands at 51 kDa, 45–47 kDa, and 18 kDa have been previously identified as vicilin subunits [29,41], with vicilin primarily distributed between 49 kDa and 14 kDa. Notably, convicilin was only slightly observed in the control sample, appearing at about 70 kDa in both the bulk and supernatant (Figure 2 and Figure 3).

Additionally, heat treatment leads to a reduction in the intensity of the legumin and vicilin bands, specifically in the ranges of 49–62 kDa and 38–49 kDa, respectively. Notably, the vicilin band around 40 kDa showed a significant decrease after heat treatment at both 4% and 8% concentrations (Figure 2 and Figure 3). The relatively low heat stability observed at a 4% protein concentration, a pH of 6.8, and a shear rate of 100 s^−1^ is also reflected in the PAGE results. In particular, the intensity of legumin A-B 2 and certain vicilin proteins in the protein dispersion at 4% concentration and a pH of 6.8 exhibited lower intensity at 100 s^−1^ compared to 1000 s^−1^ (Figure 2A).

Aligned with our previous study, all protein dispersions with high solubility contained aggregates appearing in the wells for both bulk and serum samples [8] (Figure 2 and Figure 3). In this current study, these large protein complexes showed greater intensity at a pH of 7.5 with an 8% concentration compared to other conditions. Moreover, at a 4% concentration, the soluble aggregates on the top of the bands were more pronounced than at a pH of 7.5. In contrast, at an 8% concentration, aggregates were more apparent at a pH of 7.5 than at a pH of 6.8. This finding will be further confirmed by reducing PAGE. As the stacking of aggregates decreased under reducing conditions, it suggests that some aggregates are held together by covalent bonds. This also indicates that the molecular behaviour of legumin and vicilin may contribute to the formation of a novel protein complex, which plays a critical role in the functionality of the faba bean protein isolate.

## 4. Discussion

Faba bean proteins consist of four classes: globulins, albumins, glutelins, and prolamins [42]. Due to their abundance, globulins play a vital role in the functional performance of faba bean protein isolate under various processing conditions. They are mainly classified [43] into two primary types based on their sedimentation coefficient: legumin (11S) and vicilin (7S) [44]. Legumins constitute the major class of globulins in faba beans, existing as hexameric assemblies (320–400 kDa). Each subunit comprises an acidic (α) and a basic (β) polypeptide chain that are interconnected by disulphide bonds [42]. Vicilin exists in a trimeric structure (145–190 kDa). They are often deficient in sulphur-containing amino acids like cysteine [45]. The third globulin of faba bean protein is convicilin, with a molecular weight of 52–99 kDa, which has been regarded as belonging to vicilin [46]. The protein profile of native FBPI as determined in our study (Figure 2 and Figure 3) aligns with previous studies [11,47].

In the current context, the solubility of the control FBPI dispersions was high at both protein concentrations. The experiments were performed under the optimal conditions identified earlier [8], resulting in all samples demonstrating solubility levels above 97% (Table 1). This outcome is consistent with the results of our prior research [8]. Similarly, the solubility of hemp seed protein isolate was significantly enhanced by hot treatment at neutral pH, which facilitated the dissociation of protein subunits, unravelled the tertiary structure, and exposed hydrophobic groups [12,22]. At the preparation temperature in the current study, hydrogen and ionic attractions are lowered, causing dissociation of the multimeric proteins into their subunit components or monomer units [8]. In this sense, moderate heating can enhance the hydration layer surrounding the protein, thereby improving its solubility (Table 1).

Furthermore, the heat stability of all FBPI dispersion samples remained over 95% under the examined conditions (Table 1). As shown by protein partitioning of the supernatant (Figure 2 and Figure 3), convicilin and some vicilin were largely absent after heat treatment irrespective of the shear, which may indicate their involvement in denaturation and aggregation, and consequently, precipitation upon centrifugation. As the SDS-PAGE results revealed (Figure 2 and Figure 3), there were more aggregates in the wells of the SDS-PAGE gels in the supernatant of the FBPI sample after heating. Under centrifugation conditions, these aggregates remained soluble but resolved under reducing conditions, implying the involvement of covalent bonding in their formation (Figure 2C,D and Figure 3C,D). Vicilin and convicilin are related and share some features, although there are some important differences. Vicilin contains 14 cysteine residues, while convicilin has only 2, in positions 24 and 407 [48]. They both have core domains made up of β-sheets and α-helical structures. Considering that these proteins form higher-order structures, it is expected that they show a certain level of aggregation, as illustrated by the FTIR analysis (Table 3). It seems that the levels of α-helical structure remained stable during shearing and heating and even increased at a pH of 7.5 and high shear (Table 3). The native β-sheets were most affected at a pH of 6.8, appearing to shift towards higher wavenumbers, which indicates the formation of intramolecular antiparallel β-sheet structures [29]. Increasing the protein concentration or pH resulted in the formation of aggregates that mainly contained parallel β-sheets (Table 3). It is well known that β-sheet aggregates are held together by hydrogen bonds, which are weak interactions and can therefore be influenced by processing conditions. While these interactions are affected by temperature [8], they may also be impacted by shear.

Previous studies have stated that the aggregates generated under heat can be fragmented by the applied shear [29,49,50]. The non-covalent bonds among legumin and vicilin molecules may be disrupted by shearing forces, leading to the dissociation of the protein aggregates or complexes. In particular, the protein structure is already destabilised under heat treatment. This combination of heat and shear facilitates the breaking of protein associations, likely resulting in the fragmentation of vicilin and legumin multimers [28,51,52]. The enhanced exposure of hydrophobic regions in globulins, combined with the shearing force, subsequently results in the fragmentation of soluble aggregates into smaller oligomeric forms [53,54]. Notably, the heat stability of FBPI was greater at a shearing rate of 1000 s^−1^ with the smallest particle size, regardless of pH and concentration (Table 1 and Table 2). Therefore, the combination of heat treatment and a specific shearing rate can disrupt certain soluble aggregates, thereby improving thermal stability.

As the dominant components of faba bean protein isolate, changes in the secondary structure of the 7S and 11S globulins are linked to the conformational structure and functionality of FBPI [13,17,38,47,55]. In the present study, protein concentration played an important role in governing the proteins’ conformation and thus, functionality during thermal processing [16,28]. Specifically, the particle size at 4% protein content was smaller than that of 8% pre- and post-heat treatment (Table 2). As the SDS-PAGE results show (Figure 2 and Figure 3), the increasing protein concentration of the FBPI dispersion generated more soluble aggregates in the supernatant. It is well known that greater protein concentration increases the likelihood of collisions and interactions among protein molecules, leading to the generation of more and larger aggregates [45,56]. Even under quiescent conditions, the contribution of intramolecular β-sheets was greater at 8%, indicating weak attractions among β-sheets. These interactions were influenced by shear, as β-sheets appeared to transition from an antiparallel to a parallel conformation (Table 3). This could be attributed to a loosely folded complex among legumin and vicilin molecules with high conformational flexibility [57,58].

Previous studies have shown that pH also plays a vital role in the formation of soluble aggregates of FBPI during heat processing, as it alters charge distribution and the cationic environment [20,25,59]. This effect is primarily due to pH-induced changes in the charge distribution of protein molecules, as observed in our study (Table 2), which in turn influence protein electrostatic interactions. Typically, there are more collisions and interactions among protein molecules at a pH of 6.8 under high shearing rate or concentration. This is reflected by larger particle sizes (Table 2) and more aggregates in the well of the PAGE (Figure 2A and Figure 3A). At low shearing rates and 4% protein concentration, the protein molecules appear to maintain their core domains and fold within the protein, as confirmed by the high proportion of β-sheet elements and increased intramolecular aggregates (Table 3). When shearing is increased to 1000 s^−1^, the proportion of β-turns significantly rises, suggesting that aggregate formation is accompanied by a loss of β-sheet elements [37]. When the concentration increases to 8%, interactions among proteins are strengthened, resulting in stronger electrostatic attractions. With the combination of high shearing rate and heat, certain hydrogen bonds in the protein are diminished [60]. These protein fractions then incorporate H^+^ from the solvent and form new hydrogen bonds, which facilitate further aggregation. As previous studies have demonstrated, convicilin (7S) and legumin (11S) contain high levels of β-sheet elements [51]. Therefore, the soluble aggregates formed at a pH of 6.8, likely driven by intermolecular interactions, are probably related to the dissociation and conformation of convicilin and legumin.

On the contrary, at a pH of 7.5, the surrounding OH^−^ ions neutralise the hydrogen bonds, contributing to the dissociation of the protein subunits [22]. In comparison to a pH of 6.8, intramolecular interactions appeared to predominate at each concentration level (Table 3). Similar results have been reported previously, showing that heating at alkaline pH results in the dissociation of large protein aggregates into smaller, soluble aggregates [28,29,30,40]. Additionally, the interactions are also influenced by shearing, since the aggregates generated at 8% protein concentration were disrupted by the shearing force, as indicated by the loss of both inter- and intramolecular aggregates. It is well known that shear may impact the properties of proteins, especially in conjunction with other factors. As higher pH levels lead to increased repulsive forces that reduce hydrogen bonding, the weak bonds susceptible to shearing stress can be broken, particularly affecting hydrophobic interactions [33]. Shear force can mechanically disentangle these structures, surpassing the van der Waals forces that hold hydrophobic clusters of globulins together. As a result, the hydrogen bonds weakened at a pH of 7.5 and the hydrophobic interactions disrupted by shear cause fewer aggregates to form and result in smaller aggregate sizes.

When solid particles like protein multimers flow, they rotate steadily, but anisometric particles do not. When fluid velocity exceeds a threshold, laminar flow turns turbulent, characterised by chaotic streamlines and rapid fluctuations in velocity and direction. Folded proteins are marginally stable and easily disrupted by environmental changes, such as increased temperature or changes in solvent quality. Their stability results from small, cooperative interactions involving attraction, repulsion, conformational freedom, hydration waters, and protein–solvent interactions [61,62].

Entropy, through side-chain flexibility and water organisation, balances with enthalpy to maintain native protein stability [63], which affects the slight free energy difference between native and denatured states [64]. Folding reduces entropy and hydration, thereby increasing free energy. Meanwhile, hydrogen bonds contribute to a negative enthalpy change, which can lead to aggregation. It appears that the shear may have governed the extent of aggregation in the current study.

Furthermore, it is unclear whether legumin, a large hexametric protein, would be substantially affected by shear, given that the structural changes are accompanied by significant fluctuations in the proportion of β-sheet structures (Table 3), with substantial improvements in solubility (*p* > 0.05) and heat stability (*p* < 0.05) (Table 1). Legumin is poorly soluble due to the presence of a substantial proportion of hydrophobic amino acids in its structure and readily engages in aggregation at elevated temperatures [65]. It also contains a significant proportion of beta sheets in its secondary structure, ranging from 31% to 47% [37].

As discussed earlier, variations in processing conditions such as protein concentration, pH, and shear rates can lead to the formation of different types of aggregates during thermal processing. These aggregates may influence the functional properties of faba bean proteins, including gelation, emulsification, and foaming. For instance, at 8% protein concentration and a pH of 6.8, a high proportion of β-sheet structures forms, along with increased 11S protein and larger aggregates, which are essential for gelation [51]. Conversely, at a pH of 7.5 with high shear rates, aggregate formation is reduced, resulting in more flexible protein structures that enhance electrostatic repulsion and surface activity of globulins [31,53,54], which are beneficial for emulsification and foaming. The results suggest that careful selection of process parameters like protein concentration and pH can produce a stable protein system with the desired particle size that remains dispersed and kinetically stable over time. For example, smaller particles at pH of 7.5 with 4% protein indicate better dispersion [20,59]. Additionally, zeta potential measurements provide insight into colloidal stability; a more negative zeta potential indicates stronger electrostatic repulsion and improved stability during processing [31]. In this study, the high zeta potential at a pH of 7.5 reflects greater electrostatic repulsion, along with a greater contribution of β-sheets, which may lead to more stable emulsions and potentially smaller droplet sizes. These parameters are critical in product formulation, as they help optimise processing conditions to achieve specific product properties and develop targeted products.

## 5. Conclusions

The selected processing conditions, including protein concentration, pH, and applied shear, all influenced the solubility and heat stability of FBPI dispersions. Initial solubility was only slightly affected by the concentration and pH. Heating under shear resulted in the formation of soluble aggregates, mainly stabilised by covalent bonds. Vicilin and convicilin appeared to be the main proteins involved in these interactions. Low shear (100 s^−1^) had a minor effect on heat stability, but a pH of 7.5 significantly improved it by enabling dissociation of the aggregates, leading to greater solubility, likely due to enhanced electrostatic repulsions. Increasing shear to 1000 s^−1^ further improved heat stability regardless of other processing conditions. These improvements stem from conformational changes that primarily reduced hydrogen bonding and led to protein restructuring upon shear. This study shows that dissociating and restructuring legumin and vicilin can produce various new structures, which then affect the heat stability of faba bean protein isolate. Some structural features suggest improved surface activity, but this has not been confirmed yet. Gaining a comprehensive understanding of protein behaviour during thermal processing can help develop higher-quality protein products in the future. Nevertheless, more research is needed to better understand these mechanisms and to clarify the molecular activities of FBPI, especially regarding ionic strength and digestibility.

## Figures and Tables

**Table 1:** Solubility and heat stability of FBPI dispersed in modified solution conditions and subjected to shearing during heating at 95 °C for 30 min with shearing rate of 100 s^−1^ or 1000 s^−1^.

Protein Dispersion		Solubility (%)	Heat Stability (%)	
Protein Concentration	pH	Control	Shear Rate	
			100 s^−1^	1000 s^−1^
4	6.8	99.2 ^A^	95.6 ^Aa^	101.7 ^Cc^
	7.5	97.9 ^A^	98.8 ^Bb^	101.6 _Cc_
8	6.8	99.3 ^A^	97.9 ^Bb^	101.0 ^Cc^
	7.5	100.8 ^A^	102.2 ^Cc^	103.1 ^Cc^
SEM		0.9	1.2	

Values are means of at least 4 independent observations (n ≥ 4). The means in a column with different upper-case superscript letters and in a row with different small-case superscript letters differ significantly (*p* < 0.05). SEM—standard error of the mean.

**Table 2:** Particle size and ζ-potential of various FBPI dispersions, both bulk and supernatants, in modified solution conditions that underwent shearing during heating at 95 °C for 30 min, with shearing rates of 100 or 1000 s^−1^.

Protein Dispersion		Particle Size (nm)						Zeta Potential (mV)					
Concentration	pH	Bulk			Serum			Bulk			Serum		
		Control	Heated 100 s^−1^	Heated 1000 s^−1^	Control	Heated 100 s^−1^	Heated 1000 s^−1^	Control	Heated 100 s^−1^	Heated 1000 s^−1^	Control	Heated 100 s^−1^	Heated 1000 s^−1^
4	6.8	491 ^Ab^	262 ^Bc^	273 ^Bc^	438 ^Bb^	254 ^Bc^	247 ^Bc^	−21.4 ^A1^	−21.6 ^A1^	−22.0 ^A1^	−21.2 ^A1^	−20.9 ^A1^	−21.1 ^A1^
	7.5	195 ^Bc^	165 ^Cc^	161 ^Cc^	198 ^Bc^	162 ^Cc^	161 ^Cc^	−23.7 ^B1^	−26.7 ^C1^	−26.0 ^C1^	−24.3 ^B1^	−25.7 ^C1^	−22.9 ^A1^
8	6.8	754 ^Aa^	672 ^Aa^	457 ^Ab^	575 ^Ab^	467 ^Ab^	413 ^Ab^	−21.8 ^A1^	−23.2 ^B2^	−23.4 ^B2^	−21.0 ^A1^	−20.7 ^A1^	−21.7 ^A1^
	7.5	232 ^Bc^	303 ^Bc^	211 ^Bc^	235 ^Bc^	291 ^Bc^	209 ^Bc^	−32.5 ^D2^	−36.7 ^E3^	−36.6 ^E3^	−31.2 ^C2^	−33.2 ^D2^	−32.1 ^C2^
	SEM	23.7						0.3					

Values are means of at least 6 independent observations (n ≥ 6). The means in a column with different upper-case letter superscript and in a row with different lower-case letter superscripts or numbers differ significantly (*p* < 0.05) for a particular measured parameter (particle size, zeta potential).

**Table 3:** The proportion of different secondary structural elements in the Amide I region of faba bean protein isolate dispersions subjected to a heat treatment (95 °C 30 min) and shearing (100 or 1000 s^−1^). The controls were not heated or sheared.

pH	Concentration (*w*/*w* %)	Shearing Rate (s^−1^)	Heat Treatment	Intermolecular β-Sheets Aggregates (IEM β-S)	Native β-Sheet	Random Coil (RC)	α-Helix (AH)	β-Turn	Intramolecular Antiparallel β-Sheet Aggregates (IEM β-S)	Aggregated β-Sheets (A β-S)
	Wavenumber (cm^−1^)			1620–1630	1630–1645	1646–1650	1651–1660	1661–1675	1675–1690	1691–1700
6.8	4	Control	No	20.4 ± 3.0 ^A^	25.0 ± 2.9 ^A^	9.6 ± 1.0 ^A^	7.5 ± 0.6 ^B^	12.0 ± 1.7 ^BC^	6.9 ± 0.9 ^F^	16.8 ± 1.0 ^A^
		100	Heated	15.9 ± 1.0 ^BC^	24.6 ± 1.6 ^A^	6.3 ± 1.5 ^A^	8.6 ± 1.0 ^AB^	12.7 ± 1.0 ^BC^	10.3 ± 1.8 ^EF^	15.8 ± 1.3 ^AB^
		1000	Heated	12.8 ± 1.2 ^CD^	8.1 ± 0.5 ^E^	8.5 ± 0.6 ^A^	7.8 ± 0.4 ^B^	21.5 ± 2.8 ^A^	12.6 ± 0.3 ^CD^	12.2 ± 1.9 ^CD^
	8	Control	No	13.5 ± 1.0 ^BC^	12.4 ± 1.1 ^CD^	9.2 ± 0.8 ^A^	10.3 ± 2.8 ^AB^	10.5 ± 1.1 ^C^	16.7 ± 1.7 ^AB^	12.2 ± 1.9 ^CD^
		100	Heated	15.3 ± 0.6 ^BC^	9.2 ± 0.1 ^DE^	8.4 ± 0.4 ^A^	9.8 ± 0.3 ^AB^	13.1 ± 0.1 ^BC^	14.0 ± 1.8 ^BC^	9.2 ± 1.6 ^E^
		1000	Heated	17.3 ± 1.9 ^AB^	9.0 ± 0.5 ^DE^	8.9 ± 0.9 ^A^	10.1 ± 0.7 ^AB^	13.7 ± 0.5 ^BC^	11.6 ± 2.3 ^DE^	8.7 ± 1.4 ^E^
7.5	4	Control	No	12.1 ± 1.2 ^D^	15.9 ± 3.3 ^B^	8.2 ± 0.6 ^A^	8.7 ± 1.8 ^AB^	12.8 ± 1.0 ^BC^	19.7 ± 1.0 ^A^	12.1 ± 1.4 ^CD^
		100	Heated	13.9 ± 1.5 ^BC^	12.8 ± 0.7 ^CD^	8.6 ± 0.2 ^A^	9.0 ± 0.5 ^AB^	12.7 ± 0.8 ^BC^	18.1 ± 1.0 ^A^	11.3 ± 1.2 ^CD^
		1000	Heated	16.7 ± 1.2 ^AB^	14.2 ± 0.9 ^BC^	8.8 ± 0.5 ^A^	8.1 ± 1.1 ^B^	12.8 ± 0.7 ^BC^	14.1 ± 0.7 ^BC^	8.0 ± 0.4 ^F^
	8	Control	No	12.8 ± 0.4 ^CD^	10.7 ± 0.8 ^DE^	7.5 ± 0.6 ^A^	10.0 ± 0.6 ^AB^	12.9 ± 0.5 ^BC^	17.5 ± 1.7 ^AB^	14.1 ± 1.2 ^BC^
		100	Heated	17.1 ± 1.7 ^AB^	12.6 ± 2.5 ^CD^	6.6 ± 0.8 ^A^	10.3 ± 1.5 ^AB^	11.1 ± 1.6 ^BC^	14.1 ± 1.3 ^BC^	10.1 ± 1.1 ^DE^
		1000	Heated	12.6 ± 0.7 ^CD^	12.4 ± 2.0 ^CD^	7.1 ± 0.4 ^A^	12.1 ± 1.0 ^A^	14.3 ± 0.8 ^B^	11.7 ± 1.3 ^DE^	13.8 ± 1.8 ^BC^

Values are mean ± SD. Means with the same superscripts in a column did not differ significantly (*p* > 0.05). The means in a column with different superscript uppercase letters differ significantly (*p* < 0.05).

## Data Availability

The original contributions presented in this study are included in the article; further inquiries can be directed to the corresponding author.

## References

[1] Ganesan K., Xu B. (2017). Polyphenol-Rich Dry Common Beans (*Phaseolus vulgaris* L.) and Their Health Benefits. Int. J. Mol. Sci..

[2] Kumar A., Agarwal D.K., Kumar S., Reddy Y.M., Chintagunta A.D., Saritha K., Pal G., Kumar S.J. (2019). Nutraceuticals derived from seed storage proteins: Implications for health wellness. Biocatal. Agric. Biotechnol..

[3] Aune D., Chan D.S.M., Lau R., Vieira R., Greenwood D.C., Kampman E., Norat T. (2011). Dietary fibre, whole grains, and risk of colorectal cancer: Systematic review and dose-response meta-analysis of prospective studies. BMJ.

[4] Clemente A., Olias R. (2017). Beneficial effects of legumes in gut health. Curr. Opin. Food Sci..

[5] Sharma G., Srivastava A.K., Prakash D. (2011). Phytochemicals of nutraceutical importance: Their role in health and diseases. Pharmacology.

[6] Oliete B., Potin F., Cases E., Saurel R. (2018). Modulation of the emulsifying properties of pea globulin soluble aggregates by dynamic high-pressure fluidization. Innov. Food Sci. Emerg. Technol..

[7] Chao D., Aluko R.E. (2018). Modification of the structural, emulsifying, and foaming properties of an isolated pea protein by thermal pretreatment. CyTA—J. Food.

[8] Yu R., Huppertz T., Vasiljevic T. (2024). Impact of Reconstitution Conditions on the Solubility of Faba Bean Protein Isolate. Foods.

[9] Sutariya S., Patel H. (2017). Effect of hydrogen peroxide on improving the heat stability of whey protein isolate solutions. Food Chem..

[10] Leonil J., Henry G., Jouanneau D., Delage M.-M., Forge V., Putaux J.-L. (2008). Kinetics of Fibril Formation of Bovine κ-Casein Indicate a Conformational Rearrangement as a Critical Step in the Process. J. Mol. Biol..

[11] Hu Y., Cheng L., Lee S.J., Yang Z. (2023). Formation and characterisation of concentrated emulsion gels stabilised by faba bean protein isolate and its applications for 3D food printing. Colloids Surf. A Physicochem. Eng. Asp..

[12] Raikos V., Ranawana V., Duthie G. (2015). Denaturation and oxidative stability of hemp seed (*Cannabis sativa* L.) protein isolate as affected by heat treatment. Plant Foods Hum. Nutr..

[13] Wang Z., Chen Z., Tan L., Tu J., Sun Y., Ye Y., Zhang S., Wu L. (2025). Impact of high-speed shear homogenization pretreatment on structure, functional characteristics, and interfacial properties: A case of Rice Glutelin. Food Chem. X.

[14] Zheng B.-A., Matsumura Y., Mori T. (1993). Conformational Changes and Surface Properties of Legumin from Broad Beans in Relation to Its Thermal Aggregation. Biosci. Biotechnol. Biochem..

[15] Mohammadian M., Madadlou A. (2018). Technological functionality and biological properties of food protein nanofibrils formed by heating at acidic condition. Trends Food Sci. Technol..

[16] Quintero J., Torres J.D., Corrales-Garcia L.L., Ciro G., Delgado E., Rojas J. (2022). Effect of the Concentration, pH, and Ca^2+^ Ions on the Rheological Properties of Concentrate Proteins from Quinoa, Lentil, and Black Bean. Foods.

[17] Bogahawaththa D., Chau N.H.B., Trivedi J., Dissanayake M., Vasiljevic T. (2019). Impact of selected process parameters on solubility and heat stability of pea protein isolate. LWT.

[18] Lin W., Barbut S. (2024). Hybrid meat batter system: Effects of plant proteins (pea, brown rice, faba bean) and concentrations (3–12%) on texture, microstructure, rheology, water binding, and color. Poult. Sci..

[19] Amat T., Assifaoui A., Buczkowski J., Silva J.V., Schmitt C., Saurel R. (2024). Interplay between soluble and insoluble protein/calcium/phytic acid complexes in dispersions of faba bean and pea protein concentrates around neutral pH. Food Hydrocoll..

[20] Ruiz G.A., Xiao W., van Boekel M., Minor M., Stieger M. (2016). Effect of extraction pH on heat-induced aggregation, gelation and microstructure of protein isolate from quinoa (*Chenopodium quinoa* Willd). Food Chem..

[21] Paulsen P.V. (2009). Isolated soy protein usage in beverages. Functional and Speciality Beverage Technology.

[22] Wang Q., Jin Y., Xiong Y.L. (2018). Heating-Aided pH Shifting Modifies Hemp Seed Protein Structure, Cross-Linking, and Emulsifying Properties. J. Agric. Food Chem..

[23] Roux L.L., Chacon R., Dupont D., Jeantet R., Deglaire A., Nau F. (2020). In vitro static digestion reveals how plant proteins modulate model infant formula digestibility. Food Res. Int..

[24] Vadivel V., Pugalenthi M. (2008). Effect of Various Processing Methods on the Levels of Antinutritional Constituents and Protein Digestibility of *Mucuna pruriens* (L.) Dc. Var. *Utilis* (wall. Ex Wight) Baker Ex Burck (velvet Bean) Seeds. J. Food Biochem..

[25] Kimura A., Fukuda T., Zhang M., Motoyama S., Maruyama N., Utsumi S. (2008). Comparison of Physicochemical Properties of 7S and 11S Globulins from Pea, Fava Bean, Cowpea, and French Bean with Those of Soybean—French Bean 7S Globulin Exhibits Excellent Properties. J. Agric. Food Chem..

[26] Utsumi S. (1992). Plant food protein engineering. Adv. Food Nutr. Res..

[27] Hall A.E., Moraru C.I. (2021). Structure and function of pea, lentil and faba bean proteins treated by high pressure processing and heat treatment. LWT.

[28] Alavi F., Chen L., Wang Z., Emam-Djomeh Z. (2021). Consequences of heating under alkaline pH alone or in the presence of maltodextrin on solubility, emulsifying and foaming properties of faba bean protein. Food Hydrocoll..

[29] Beck S.M., Knoerzer K., Sellahewa J., Emin M.A., Arcot J. (2017). Effect of different heat-treatment times and applied shear on secondary structure, molecular weight distribution, solubility and rheological properties of pea protein isolate as investigated by capillary rheometry. J. Food Eng..

[30] Nivala O., Nordlund E., Kruus K., Ercili-Cura D. (2021). The effect of heat and transglutaminase treatment on emulsifying and gelling properties of faba bean protein isolate. LWT.

[31] Keivaninahr F., Gadkari P., Benis K.Z., Tulbek M., Ghosh S. (2021). Prediction of emulsification behaviour of pea and faba bean protein concentrates and isolates from structure–functionality analysis. RSC Adv..

[32] Dissanayake M., Liyanaarachchi S., Vasiljevic T. (2012). Functional properties of whey proteins microparticulated at low pH. J. Dairy Sci..

[33] Mediwaththe A., Bogahawaththa D., Grewal M.K., Chandrapala J., Vasiljevic T. (2018). Structural changes of native milk proteins subjected to controlled shearing and heating. Food Res. Int..

[34] Sharan S., Zotzel J., Stadtmüller J., Bonerz D., Aschoff J., Olsen K., Rinnan Å., Saint-Eve A., Maillard M.-N., Orlien V. (2022). Effect of industrial process conditions of fava bean (*Vicia faba* L.) concentrates on physico-chemical and functional properties. Innov. Food Sci. Emerg. Technol..

[35] Meng G.-T., Ma C.-Y. (2001). Fourier-transform infrared spectroscopic study of globulin from Phaseolus angularis (red bean). Int. J. Biol. Macromol..

[36] Tang C.-H., Sun X., Foegeding E.A. (2011). Modulation of physicochemical and conformational properties of kidney bean vicilin (phaseolin) by glycation with glucose: Implications for structure–function relationships of legume vicilins. J. Agric. Food Chem..

[37] Carbonaro M., Maselli P., Dore P., Nucara A. (2008). Application of Fourier transform infrared spectroscopy to legume seed flour analysis. Food Chem..

[38] Wang J., Liu H., Ren G. (2014). Near-infrared spectroscopy (NIRS) evaluation and regional analysis of Chinese faba bean (*Vicia faba* L.). Crop J..

[39] Deng G., Rodríguez-Espinosa M.E., Yan M., Lei Y., Guevara-Oquendo V.H., Feng X., Zhang H., Deng H., Zhang W., Samadi (2020). Using advanced vibrational molecular spectroscopy (ATR-Ft/IRS and synchrotron SR-IMS) to study an interaction between protein molecular structure from biodegradation residues and nutritional properties of cool-climate adapted faba bean seeds. Spectrochim. Acta Part A Mol. Biomol. Spectrosc..

[40] Berrazaga I., Bourlieu-Lacanal C., Laleg K., Jardin J., Briard-Bion V., Dupont D., Walrand S., Micard V. (2020). Effect of protein aggregation in wheat-legume mixed pasta diets on their in vitro digestion kinetics in comparison to “rapid” and “slow” animal proteins. PLoS ONE.

[41] Peng W., Kong X., Chen Y., Zhang C., Yang Y., Hua Y. (2016). Effects of heat treatment on the emulsifying properties of pea proteins. Food Hydrocoll..

[42] Martineau-Côté D., L’Hocine L., Tuccillo F., Wanasundara J.P.D., Stoddard F.L., Nadathur S., Wanasundara J.P.D., Scanlin L. (2024). Chapter 8—Faba Bean as a Sustainable Plant Protein Source. Sustainable Protein Sources.

[43] Danielsson C.E. (1949). Seed globulins of the Gramineae and Leguminosae. Biochem. J..

[44] Mundi S., Aluko R.E. (2012). Physicochemical and functional properties of kidney bean albumin and globulin protein fractions. Food Res. Int..

[45] Tang Q., Roos Y.H., O’Sullivan M., Miao S. (2025). A comparative study on gelation behaviours of lentil-dairy binary protein gels treated by heat and microbial transglutaminase. Food Hydrocoll..

[46] Lam A.C.Y., Can Karaca A., Tyler R.T., Nickerson M.T. (2018). Pea protein isolates: Structure, extraction, and functionality. Food Rev. Int..

[47] Liu C., Damodaran S., Heinonen M. (2019). Effects of microbial transglutaminase treatment on physiochemical properties and emulsifying functionality of faba bean protein isolate. LWT.

[48] Dong C., Zhao J., Wang L., Jiang J. (2024). New insights into the cross-linking between myosin and alkali-treated pea protein by transglutaminase under low ionic conditions: Contribution of legumin and vicilin fractions. Food Hydrocoll..

[49] Sanchez-Monge R., Lopez-Torrejón G., Pascual C.Y., Varela J., Martin-Esteban M., Salcedo G. (2004). Vicilin and convicilin are potential major allergens from pea. Clin. Exp. Allergy.

[50] Shevkani K., Singh N., Kaur A., Rana J.C. (2015). Structural and functional characterization of kidney bean and field pea protein isolates: A comparative study. Food Hydrocoll..

[51] Mession J.-L., Chihi M.L., Sok N., Saurel R. (2015). Effect of globular pea proteins fractionation on their heat-induced aggregation and acid cold-set gelation. Food Hydrocoll..

[52] O’Kane F.E., Happe R.P., Vereijken J.M., Gruppen H., van Boekel M.A.J.S. (2004). Characterization of Pea Vicilin. 1. Denoting Convicilin as the α-Subunit of the *Pisum* Vicilin Family. J. Agric. Food Chem..

[53] Zhang J., Liu Q., Chen Q., Sun F., Liu H., Kong B. (2022). Synergistic modification of pea protein structure using high-intensity ultrasound and pH-shifting technology to improve solubility and emulsification. Ultrason. Sonochem..

[54] Taha A., Hu T., Zhang Z., Bakry A.M., Khalifa I., Pan S., Hu H. (2018). Effect of different oils and ultrasound emulsification conditions on the physicochemical properties of emulsions stabilized by soy protein isolate. Ultrason. Sonochem..

[55] Choi S.-M., Ma C.-Y. (2007). Structural characterization of globulin from common buckwheat (*Fagopyrum esculentum* Moench) using circular dichroism and Raman spectroscopy. Food Chem..

[56] Shrestha S., van’t Hag L., Haritos V., Dhital S. (2023). Rheological and textural properties of heat-induced gels from pulse protein isolates: Lentil, mungbean and yellow pea. Food Hydrocoll..

[57] Qu W., Zhang X., Chen W., Wang Z., He R., Ma H. (2018). Effects of ultrasonic and graft treatments on grafting degree, structure, functionality, and digestibility of rapeseed protein isolate-dextran conjugates. Ultrason. Sonochem..

[58] Wang Q., Johnson J.L., Agar N.Y., Agar J.N., Weissman J.S. (2008). Protein aggregation and protein instability govern familial amyotrophic lateral sclerosis patient survival. PLoS Biol..

[59] Hu Y., Cheng L., Gilbert E.P., Lee S.J., Yang Z. (2024). Impact of thermosonication at neutral pH on the structural characteristics of faba bean protein isolate dispersions and their physicochemical and techno-functional properties. Food Hydrocoll..

[60] Li C., Tian Y., Liu C., Dou Z., Diao J. (2023). Effects of heat treatment on the structural and functional properties of *Phaseolus vulgaris* L. protein. Foods.

[61] Shen P., Ma X., Griskonyte J., Peng J., Gouzy R., Sagis L.M., Landman J. (2025). Augmentation of faba bean globulin gelation with pre-aggregation. Food Hydrocoll..

[62] Fenimore P.W., Frauenfelder H., McMahon B.H., Parak F.G. (2002). Slaving: Solvent fluctuations dominate protein dynamics and functions. Proc. Natl. Acad. Sci. USA.

[63] Scharnagl C., Reif M., Friedrich J. (2005). Stability of proteins: Temperature, pressure and the role of the solvent. Biochim. Biophys. Acta.

[64] Walstra P., Dickinson E., Miller R. (2001). Effects of agitation on proteins. Food Colloids: Fundamentals of Formulation.

[65] Oluwajuyitan T.D., Aluko R.E. (2024). Structural and functional properties of fava bean legumin and vicilin protein fractions. Int. J. Food Sci. Technol..

